# Hip pain in adolescents with cerebral palsy: a population‐based longitudinal study

**DOI:** 10.1111/dmcn.14782

**Published:** 2021-01-03

**Authors:** Selma Mujezinović Larsen, Kjersti Ramstad, Terje Terjesen

**Affiliations:** ^1^ Department of Clinical Neurosciences for Children Rikshospitalet Oslo University Hospital Oslo Norway; ^2^ University of Oslo Oslo Norway; ^3^ Department of Orthopaedic Surgery Rikshospitalet Oslo University Hospital Oslo Norway

## Abstract

**Aim:**

To investigate the prevalence, characteristics, and risk factors of hip pain in adolescents with cerebral palsy (CP) and compare the findings with those of the same individuals 5 years earlier.

**Method:**

Sixty‐seven adolescents (28 females, 39 males; mean age 14y 7mo; SD 1y 5mo; range 12–17y) with bilateral CP, in Gross Motor Function Classification System (GMFCS) levels III to V enrolled in a CP surveillance programme were assessed for hip pain. Their caregivers responded to the questions on the intensity and frequency of hip pain from the Child Health Questionnaire (CHQ) (transformed to CHQ hip pain score; 100 indicates no pain). Interference of hip pain with daily activities and sleep was recorded on numeric rating scales. Hip displacement was measured radiographically by the migration percentage.

**Results:**

Twenty‐eight participants had 44 painful hips. Their mean CHQ hip pain score was 40 (SD 21.4; range 10–80). Independent risk factors for hip pain, low CHQ hip pain score, and interference with sleep were severe hip subluxation (migration percentage 50–89%) and GMFCS level V. A migration percentage of 50% to 89% was the only independent risk factor for interference with daily activities. Over 5 years, the number of participants with hip pain increased from 18 to 28, while the mean migration percentage of the most displaced hip was unchanged.

**Interpretation:**

Our CP hip surveillance programme did not protect the participants against increasing prevalence of hip pain during adolescence. We suggest that surveillance programmes for CP should include guidelines on the characteristics and management of hip pain.

**What this paper adds:**

- Hip pain prevalence increased in adolescents over a 5‐year period in a cerebral palsy surveillance programme.
- Risk factors for hip pain were Gross Motor Function Classification System level V and severe hip subluxation.

## What this paper adds

- Hip pain prevalence increased in adolescents over a 5‐year period in a cerebral palsy surveillance programme.
- Risk factors for hip pain were Gross Motor Function Classification System level V and severe hip subluxation.

**Abbreviations**
CHQ: Child Health Questionnaire
CPOP: Cerebral Palsy Follow‐Up Program
ITB: Intrathecal baclofen

Seventy‐five percent of children and adolescents with cerebral palsy (CP) have pain, most frequently located in the lower limbs.^1^, ^2^ In a hospital‐based study, physicians identified hip displacement as the most frequent primary source of severe pain in children and adolescents with CP. This accounted for 24% of the causes of pain that had prevented participation in activities, confirming the clinical significance of hip pain.^3^ In accordance with this, several countries have developed surveillance programmes for children with CP, aiming to monitor hip displacement, avoid hip dislocation, and prevent hip pain.^4^, ^5^

Hip displacement is usually measured radiographically by the migration percentage.^6^ A few studies have explored the association between hip pain and increasing migration percentage in CP, but different age groups and strategies to assess hip pain make comparison of the results difficult.^7^, ^8^, ^9^, ^10^ In non‐ambulatory young adults, the prevalence of hip pain increased with increasing migration percentage.^7^ In a study of non‐ambulatory children aged 7 to 12 years, hip pain was found to be significantly associated with severe hip displacement, while mild and moderate displacement did not influence the occurrence of hip pain. Hip pain occurred in 60% of hips with a migration percentage of ≥50%.^10^ Another study of children aged 4 to 16 years confirmed an association between hip pain and hip displacement.^11^ However, no association between hip pain and migration percentage was found in 18 participants aged 2 to 21 years.^9^ Thus, further studies on the relationship between hip pain and hip displacement are warranted.

A Swedish register‐based study on pain in children and adolescents with CP found that pain, even when repeatedly reported during follow‐up, was largely neglected in the corresponding medical records.^12^ This indicates that there is a knowledge gap between recording pain and providing adequate pain management in CP‐surveillance programmes.

The aims of this study were to assess the prevalence of hip pain over a 5‐year period in a population‐based cohort of adolescents with CP and to investigate the characteristics and risk factors of hip pain and the interference of such pain with daily activities and sleep.

## METHOD

The present study is a longitudinal, population‐based study of non‐ambulatory adolescents, enrolled in the Norwegian Cerebral Palsy Follow‐Up Program (CPOP),^13^ born from 2002 to 2006, with bilateral CP and living in south‐eastern Norway. Data on CP diagnosis^14^ according to the Surveillance of Cerebral Palsy in Europe^15^ and ambulation according to Gross Motor Function Classification System (GMFCS)^16^ was retrieved from CPOP.

The study was approved by the Regional Ethics Committee, REC South East (reference 2012/2258 REK). Informed written consent was obtained for 77 participants recruited to the previous study in the period 2013 to 2014.^10^ Six participants were lost to follow‐up: four had died, one had moved out of Norway, and one had left the CPOP. Thus, 71 participants received a postal invitation to participate in the present data collection during 2019, of whom 67 participated (94%).

### Assessment

Information on hip pain was collected from primary caregivers during a structured telephone interview performed by one of the authors (SML). Hip pain was recorded as a dichotomous variable: no pain or pain. If hip pain was present, laterality was noted. Caregivers were asked: ‘What is the reason that you believe your child has pain?’ The circumstances of hip pain were explored by asking: ‘In which situations does hip pain occur?’ with an open response, which we classified in pain linked to position (long time in the same position, change of position, during personal care), provoked pain (muscle stretching, palpation, weight bearing on lower limb), and spontaneous pain (at night, dependent on temperature), as proposed by Hodgkinson et al.^7^

The questions on pain from the Child Health Questionnaire (CHQ, Norwegian version)^17^ were applied for (1) pain intensity: ‘During the last 4 weeks how much hip pain or discomfort has your child had?’ with the response alternatives ‘none, very mild, mild, moderate, severe, and very severe’; and (2) pain frequency: ‘During the last 4 weeks how often did your child have hip pain or discomfort?’ with the response alternatives ‘none of the time, once or twice, a few times, fairly often, very often, and every day or almost every day’ respectively scored 1 to 6. Scores were transformed by an algorithm into a 0 to 100 scale, where 100 indicates no pain.^18^ For the purpose of the present study, we categorized hip pain scores 10 to 30 as severe, 40 to 60 as moderate, and 70 to 90 as mild hip pain.

To assess the interference of hip pain with daily activities and sleep, the Brief Pain Inventory (Norwegian version) was utilized.^19^ The Brief Pain Inventory uses a numeric rating scale from 0 to 10 for pain interference with function, where 0 indicates no interference. The questions were modified into asking for pain interference over the last 4 weeks.

Caregivers responded if the participant was on intrathecal baclofen (ITB) therapy and other medication for spasticity and pain over the last 4 weeks, and whether hip surgery had been performed. Further details on hip surgery were available from the CPOP.

The latest radiograph of the pelvis and hip joints, taken for CPOP, were transferred to Oslo University Hospital’s Picture Archiving and Communication System (PACS; Sectra, Linköping, Sweden) after the interview. If the latest radiograph was taken before 2017, the respondent was asked to permit a new radiograph to be taken. The radiographs were enlarged for better visualization of the landmarks, and measurements were performed digitally by one of the authors (TT). Migration percentage was measured in both hips using Reimers’ method.^6^ Depending on their migration percentage, the hips were categorized as normal (migration percentage <33%), subluxated (migration percentage 33–89%), or dislocated (migration percentage ≥90%).^13^ Further, hip subluxation was categorized as mild (migration percentage 33–39%), moderate (migration percentage 40–49%), and severe (migration percentage 50–89%). Pelvic obliquity was measured as the angle between the horizontal line and the line between the lowest points of the pelvic bones on the right and left side.^20^ Further, the presence of deformities of the proximal femur (flattening or deformity of the femoral head and marked shortening or pronounced varus of the femoral neck) was assessed. Sixty‐one participants had available radiographs. Three participants had complete hip dislocation on older radiographs. As dislocation is a permanent condition when left untreated, and no hip surgery had been performed, these participants were included in the radiographic evaluation. Three participants had ended the hip surveillance programme. Thus, the radiographic evaluation included 64 of the 67 participants. The median time between radiograph and interview was 5 months before the interviews (range 26mo before the interview to 6mo after the interview on hip pain).

### Statistics

SPSS version 26 (IBM, Armonk, New York, USA) was used for the statistical analysis. A comparison of proportions was analyzed using McNemar’s test. Categorical variables were analysed with the Pearson *χ* ^2^ test and logistic regression, while continuous variables were analysed with Student’s *t*‐test, analyses of variance with Scheffe’s post hoc test, and with linear regression. For the evaluation of risk factors for hip pain and low CHQ hip pain score, each variable was initially assessed in univariable analyses. Then variables significant at the 0.05 level were included in a multivariable regression analysis. Paired sample *t*‐test was used for longitudinal analysis of migration percentage of the most displaced hip. All tests were two‐sided. Differences were considered significant when *p*<0.05.

## RESULTS

Sixty‐seven adolescents (28 females, 39 males; mean age 14y 7mo; SD 1y 5mo; range 12–17y) with bilateral CP, participated in the study. Fifty‐three participants (79%) had a predominant spastic movement disorder, while 14 had dyskinetic CP. GMFCS level distribution was: GMFCS level III 15 (22%), GMFCS level IV 17 (25%), and GMFCS level V 35 (52%). Thirty‐two participants were receiving medication for epilepsy and 15 were receiving ITB. Hip surgery had been performed in 47 participants; soft‐tissue releases in 21, and bony procedures in 26.

Hip pain was reported in 28 participants and in 44 hips. Pain was bilateral in 16 participants and unilateral in 12. Caregivers reported that 24 participants were able to self‐report on pain verbally, 31 participants expressed pain with special sounds and gestures, and 12 caregivers based their answers on observation of behavior during daily routines. Two caregivers were not able to localize pain, and their responses were noted as ‘no hip pain’.

**Figure 1:**
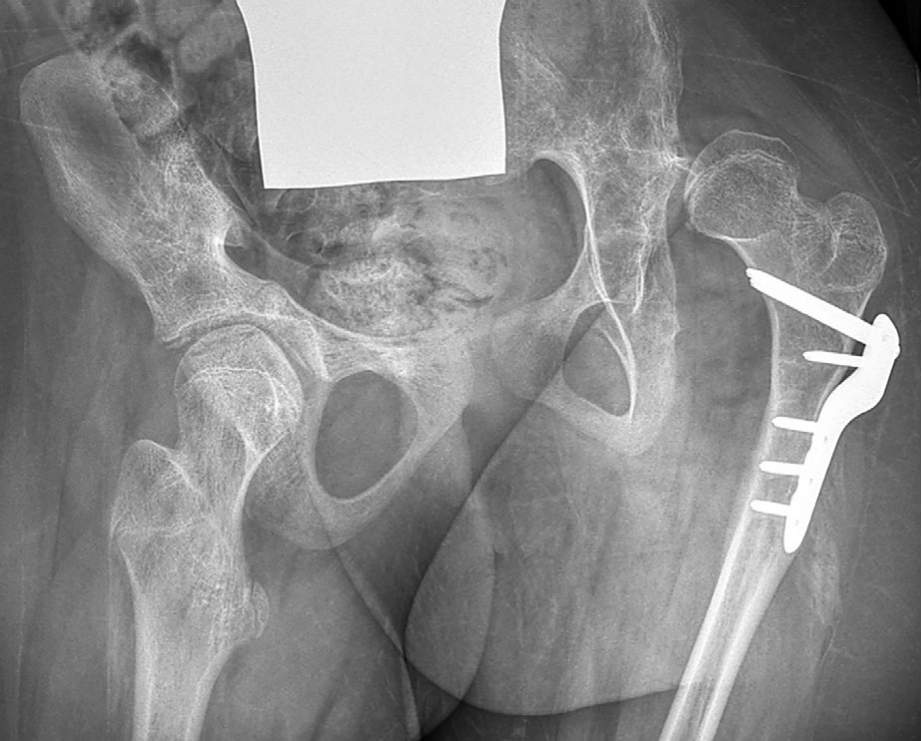
Radiograph of a 14‐year‐old female with bilateral spastic cerebral palsy in Gross Motor Function Classification System level IV, showing severe subluxation and femoral head deformity of the left hip and marked pelvic obliquity. She had unilateral severe pain in her left hip (Child Health Questionnaire hip pain score 20).

**Figure 2:**
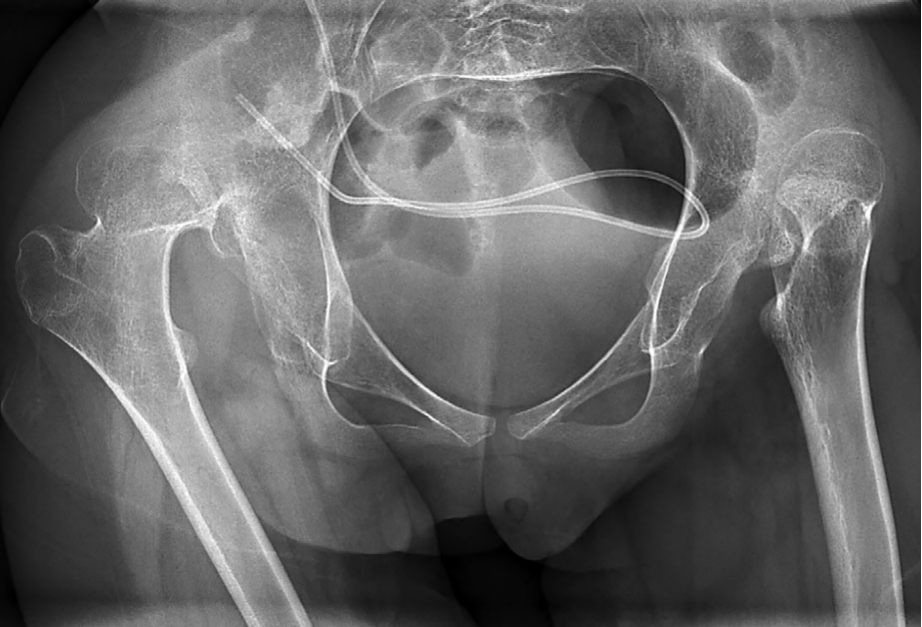
Radiograph of a 14‐year old female with bilateral spastic cerebral palsy in Gross Motor Function Classification System level V, with painless bilateral complete hip dislocation, deformity of the right femoral head, and no pelvic obliquity.

There was no significant difference between the prevalence of pain in hips with normal migration percentage and hips with mild or moderate subluxation: 31% and 21% respectively (Table 1). However, the prevalence was significantly higher (*p*=0.004) in the group of eight patients with severe subluxation (migration percentage 50–89%), where pain was present in eight of the nine subluxated hips. Five of these patients had unilateral severe subluxation (migration percentage 52–75%), unilateral pain in the subluxated hip, and pronounced pelvic obliquity ranging from 5° to 22° with the subluxated side highest (Fig. 1). In the eight hips (five patients) with complete dislocation, hip pain was present in only one hip. Three of these patients had bilateral, painless dislocation, and pelvic obliquity was <6° (Fig. 2).

**Table 1:** Association between hip pain and hip migration percentage in 128 hips of 64 adolescents with cerebral palsy

Migration %^a^	Number of hips	Hip pain		*p*
		No hip pain	Hip pain	
All hips	128	86	42	
<33	97	67	30	0.004
33–39	9	7	2	
40–49	5	4	1	
50–89	9	1	8	
90–100	8	7	1	

^a^ Three patients did not have available pelvic radiographs.

Patient‐related risk factors for hip pain are shown in Table 2. When parameters associated with hip pain were tested in multivariable logistic regression, GMFCS level V was statistically significant, whereas age was not. Migration percentage could not be computed because one of the categories contained no hips. Deformities of the proximal femur were found in 18 of the 128 hips. The plate used for fixation of femoral osteotomy had not been removed in 25 hips. Both femoral deformity and the presence of a femoral plate were significantly associated with hip pain (*p*=0.006 and *p*=0.023 respectively). When both these variables were analyzed together with migration percentage and GMFCS in multivariable logistic regression, GMFCS level V and migration percentage 50% to 89% remained as independent risk factors.

**Table 2:** Association between hip pain and possible risk factors for hip pain in 67 adolescents with cerebral palsy

Risk factor	No hip pain	Hip pain	Univariable *p*	Multivariable *p*
Age, y:mo, mean (SD)	14:11 (1:6)	14:2 (1:4)	0.018	0.202
Sex				
Female	15	13	0.514	
Male	24	15		
Predominant movement disorder				
Spastic	31	22	0.928	
Dyskinetic	8	6		
Ambulation				
GMFCS level III	13	2	0.013	0.027
GMFCS level IV	11	6		
GMFCS level V	15	20		
Hip surgery				
No	14	6	0.202	
Yes	25	22		
ITB				
No	31	21	0.664	
Yes	8	7		
Migration %, most displaced hip				
<50%	33	20	0.004^a^	
50–89%	0	6		

Data are number of participants, unless otherwise stated.

^a^ Multivariable logistic regression could not be computed because one of the categories contained no hips. GMFCS, Gross Motor Function Classification System; ITB, intrathecal baclofen.

In the 28 participants with hip pain, the mean CHQ hip pain intensity score was 3.8 (SD 0.8, range 2–6) and mean frequency score was 4.3 (SD 1.6, range 2–6). Their mean CHQ hip pain score was 40 (SD 21.4). Severe hip pain (score 10–30) was present in 13 patients, whereas 10 had moderate hip pain (score 40–60) and five had mild hip pain (score 70–90). Parameters significantly associated with low CHQ hip pain score were GMFCS level V and migration percentage 50% to 89% (Table 3). In multivariable linear regression, both GMFCS level V and migration percentage 50% to 89% remained as independent risk factors. The mean hip pain score in patients with normal migration percentage was 77 (SD 32.9) and differed significantly from a score of 33 (SD 22.5) in patients with severely subluxated hips (*p*=0.003).

**Table 3:** Association between Child Health Questionnaire (CHQ) hip pain score and clinical and radiographic variables

Variable	CHQ hip pain score	Univariable *p*	Multivariable *p*
Age		0.044	0.267
Sex			
Female	73 (33.8)	0.721	
Male	76 (32.5)		
Predominant movement disorder			
Spastic	75 (33.5)	0.849	
Dyskinetic	76 (31.3)		
Ambulation			
GMFCS level III	96 (11.2)	0.003	0.001
GMFCS level IV	82 (28.1)		
GMFCS level V	63 (35.9)		
Hip surgery			
No	85 (27.0)	0.120	
Yes	71 (34.4)		
ITB			
No	76 (32.2)	0.572	
Yes	71 (35.8)		
Migration %, most displaced hip (*n*=59)			
<50%	77 (32.1)	0.002	0.005
50–89%	33 (22.5)		

Data are mean (SD).

GMFCS, Gross Motor Function Classification System; ITB, intrathecal baclofen therapy.

The mean value of hip pain interference with daily activities was 3.1 (SD 3.1, range 0–10) and the mean interference with sleep was 1.9 (SD 2.6, range 0–9). Parameters significantly associated with high interference of hip pain with daily activities were GMFCS level V (*p*=0.030) and migration percentage 50% to 89% (*p*<0.001). In multivariable linear regression, migration percentage 50% to 89% remained as the only independent risk factor (*p*<0.001). Parameters associated with high interference of hip pain with sleep were GMFCS level V (*p*=0.003) and migration percentage 50% to 89% (*p*<0.001). In multivariable regression both factors were statistically significant (*p*=0.011 and *p*<0.001 respectively).

Apart from four patients with one circumstance of hip pain, the other 24 patients had between two and eight circumstances in which they experienced hip pain (Table S1, online supporting information). Pain linked to position included ‘long time in the same position’ in 21 patients, ‘change of position’ in 20, and ‘during personal care’ in 21. Pain was provoked by stretching in 11 patients, by palpation in three, and at weight‐bearing in six. Spontaneous pain at night was present in 16 patients and in cold weather in three.

Seven of the 28 patients with hip pain receiving ITB used additional pain medication (paracetamol and/or ibuprofen) daily. Four of these seven patients had severe hip pain. Thirteen patients received medication daily either for spasticity (six patients) or for pain (six patients), or a combination of these (one patient), while the remaining eight patients received pain medication either occasionally (two patients) or not at all (six patients).

The results were compared with the corresponding findings of the same individuals 5 years earlier (Table 4). The mean time between assessments was 5 years 1 month (range 3y 8mo–5y 11mo). The prevalence of hip pain had increased (*p*=0.041) while the mean migration percentage of the most displaced hip was unchanged (*p*=0.577). Ten participants had undergone hip surgery between the two data collections. The prevalence of bilateral hip pain had increased from four to 16 patients.

**Table 4:** Comparison between the present study and the previous study (5 years earlier) of the same cohort (67 patients)

Variables	Present study	Previous study	*p*
Age, y:mo, mean (range)	14:7 (12:0–17:0)	9:6 (7:0–12:0)	
Hip pain, participants	28 (42)	18 (27)	0.041
Hip pain, all hips^a^	44 (33)	22 (16)	0.001
CHQ hip pain score, mean (SD)	75 (33)	N/A	
Hip surgery	47 (70)	41 (61)	0.031
ITB	15 (24)	10 (15)	0.063
Migration %, most displaced hip, mean (SD)^b^	36 (24)	35 (22)	0.577

Data are *n* (%) unless otherwise stated.

^a^ Total number of hips *n*=134.

^b^ Available for 64 participants. CHQ, Child Health Questionnaire; N/A, not available; ITB, intrathecal baclofen.

## DISCUSSION

The prevalence of hip pain increased with increasing GMFCS levels from level III to V. This shows that hip pain was most frequent in non‐ambulatory participants, which is in accordance with previous studies.^2^, ^10^, ^11^ The prevalence was 35% in patients in GMFCS level IV and 57% in GMFCS level V. The corresponding rates of proxy‐reported hip pain were 28% and 44% in the SPARCLE2 study of adolescents aged 13 to 17 years^2^ and 8% and 20% in a registry‐based study from Sweden.^11^ The reason for our higher hip pain rate in GMFCS level V than that of SPARCLE2 is unclear. One obvious reason for the lower hip pain rates in the Swedish study^11^ is that patients with ITB (almost one‐sixth of the population in GMFCS levels IV and V) were excluded from the study. Our data showed that hip pain was present in about half the patients with ITB, which indicates that patients receiving ITB should be included in studies on hip pain.

Pain at any site seems to increase with increasing age in children and adolescents with CP.^2^ The prevalence of hip pain was higher in children aged 7 to 16 years than in children aged 4 to 6 years.^11^ We found no previous study where hip pain was analyzed longitudinally in the same individuals, as was done in the current study. The prevalence of hip pain increased from 27% to 42% over the 5‐year period. The prevalence was stable for GMFCS level III (13%), but increased for GMFCS levels IV and V. Since GMFCS levels were unchanged and no significant increase in mean migration percentage over the 5‐year period was seen, we have no clear explanation for the increase of hip pain in non‐ambulatory children. One possible reason could be the inclusion of questions giving the CHQ hip pain score in the present study.

Similar to the previous study,^10^ independent risk factors for hip pain were GMFCS level V and severe subluxation (migration percentage 50–89%). On both occasions, hip pain was not more frequent in hips with mild or moderate subluxation (migration percentage 33–49%) than in hips with normal migration percentage. This means that a migration percentage of <50% is of little or no clinical significance for hip pain and that other causes should be searched for. Marcström et al. reported a higher rate of pain in patients with a migration percentage of ≥40% compared with children with lower migration percentages, but the side of hip pain was not specified.^11^ In our patients, severe subluxation was associated with hip pain in all except one hip. Five of these patients had unilateral subluxation and marked pelvic obliquity, with the high side of the pelvis corresponding to the subluxated hip. All except one had undergone surgery for severe hip subluxation before the present study (one femoral osteotomy and three combined femoral and pelvic osteotomies) but had experienced relapse.

The reason for not including the hips with complete dislocation in the group with severe subluxation in the risk factor analysis for pain was that the prevalence of hip pain differed markedly between these two groups. The rate of hip pain in the group with complete dislocation was very low (one out of eight hips). However, our data must be taken with caution since this group contained only five patients. Three of these patients had painless bilateral complete dislocation. Whether this is a chance finding is difficult to know, because we found no previous study where the prevalence of hip pain in adolescents with severe subluxation and complete dislocation was compared. In non‐ambulatory adults, the prevalence of hip pain in dislocated hips in studies that were not population‐based has been reported to be 29% to 50%.^8^, ^21^, ^22^

Although the aim of hip screening is to avoid severe subluxation and complete dislocation, this aim is not always achieved. However, if not accompanied by pain or pelvic obliquity, hip dislocation does not need to be a significant problem, and the indications for major bony surgery are open to discussion, especially if the dislocation is bilateral. Two of our three patients with bilateral complete dislocation joined the CPOP at a rather late age (9y) and the parents of the third refused hip surgery. Since they had no hip pain, major hip surgery was not advisable. Two patients had complete dislocation in one hip and severe subluxation in the other. One of these patients had relapse of hip displacement after bilateral femoral osteotomies, but no reoperation was performed since he had no hip pain. The parents of the other had refused hip surgery.

Hip pain was also frequent in hips with normal migration percentages, with a rate of 31%, indicating causes other than subluxation. Two possible causes are deformity of the proximal femoral head and/or neck and a persisting femoral plate (we do not routinely remove plates used for osteosynthesis in children with CP). Both these factors were significantly associated with hip pain. In cases of pronounced femoral deformity, proximal femoral resection should be considered if the patient has significant pain. A plate can cause pain over the trochanter region, especially if the plate protrudes laterally, and removal of the plate would be the logical treatment. Patients who have undergone femoral osteotomy should routinely be asked whether they have such pain. Contractures and severe spasticity could also contribute to hip pain.

Previous studies on hip pain in children and adolescents with CP have not evaluated the characteristics and impact of such pain.^2^, ^10^, ^11^ Hip pain was moderate to severe in 23 of 28 participants, occurred in more than half the participants during sleep, and in three of four participants during changes of position and personal care. The interference of hip pain with daily activities and sleep was usually mild or moderate, with a trend towards greater interference with daily activities than with sleep.

Despite daily medication for pain and/or spasms, hip pain was still present in 20 patients, indicating that pain management had been insufficient. Pain assessment in our surveillance programme (CPOP) includes only the presence and location of pain. We suggest the inclusion of additional information on the laterality of hip pain, the CHQ hip pain score and the interference with daily activities in CP surveillance programmes and to use these parameters to develop guidelines on hip pain management.

Mild hip pain (CHQ hip pain score 70–90) should be addressed by the local care team aiming to avoid situations that provoke pain. Further, we suggest a CHQ hip pain score cut‐off at 60 (moderate hip pain) for referral to a multidisciplinary team in specialist healthcare, aiming to assess reasons for hip pain, to develop a pain management plan that includes pharmacological treatment, and to revise it regularly. According to the existing CPOP guidelines, patients with displaced hips (migration percentage ≥40%) should be referred to a multidisciplinary evaluation with an orthopaedic surgeon and a child neurologist. A recent study on non‐ambulatory patients with severe hip displacement showed that hip surgery had a good effect on hip pain.^20^ In particular, patients with unilateral subluxation combined with pelvic obliquity should be offered surgical treatment, including femoral and/or pelvic osteotomies, if their general condition allows such major surgery. In patients with painless bilateral complete dislocation, surgical treatment to relocate hips is hardly indicated.

There are several limitations to this study. First, the number of patients is relatively small. Second, hip pain was only proxy‐reported because a high proportion of participants had intellectual disabilities and/or impaired communication skills. It is difficult to find the ‘true’ prevalence of hip pain in this population because other conditions can be mistaken for hip pain. Third, the assessment was performed by a new investigator, which might have influenced the reliability of the comparison. There are also several strengths of the study. The participants were recruited from a population‐based CP surveillance programme and the same 67 individuals were reassessed for hip pain. Characteristics of hip pain, such as intensity, frequency, circumstances, and the interference of hip pain with daily activities and sleep, were performed for the first time in a population‐based study.

In conclusion, our findings confirm that hip pain is a considerable problem for non‐ambulatory children and adolescents with CP. The main risk factors were GMFCS level V and severe hip subluxation, but pain was also present in almost one‐third of non‐displaced hips. The prevalence of hip pain increased, which indicates that an action plan on hip pain management should be included in CP surveillance.

## Supporting information


**Table S1.** Data on 28 participants with hip pain

## Data Availability

The data that support the findings of this study are available from the corresponding author upon reasonable request.
