# Supplementary material for: Hip pain in adolescents with cerebral palsy: a population‐based longitudinal study
Source: Dev Med Child Neurol. 2021 Jan 3;63(5):601–7. doi: 10.1111/dmcn.14782 (PMC8048986; doi:10.1111/dmcn.14782)
Supplement: Supplementary file 1 — Table S1. Data on 28 participants with hip pain [file DMCN-63-601-s001.docx]

___________________________________________________________________________________________________________________________________________

Supplementary Table 1. Data on 28 participants with hip pain

Age, Gender GMFCS Surgery ITB Intensity Frequency CHQ Interference on Side of MP Circumstances Medication

y:mo DA Sleep hip pain MP max MP min

____________________________________________________________________________________________________________________________________________________________

12:2 F IV N N 4 6 20 0 0 B 30 23 1ac No

12:6 M V Y N 4 6 20 7 3 B 35 12 2abc 3a B Pᵒ Iᵒ

12:7 F V Y Y 2 2 80 0 0 B NA NA 2abc Pᵈ Iᵒ

12:8 M IV Y N 3 3 70 1 0 U 73 23 1c 2a 3a No

12:8 F III N N 2 2 80 0 0 B 15 13 2a Pᵒ Iᵒ

12:9 M IV Y Y 4 4 40 2 2 B 23 19 2a 3ab Pᵒ Iᵒ

13:1 F V Y N 4 6 20 0 0 B 15 7 1a No

13:2 M V Y N 4 4 40 3 6 U 48 40 2abc 3ab No

13:4 F V Y N 4 3 50 3 2 U 61 27 1b 2abc 3a B Pᵒ Nᵒ

13:4 M V Y N 5 6 10 4 4 U 30 25 2b 3a Pᵈ

13:5 F V Y N 4 6 20 7 7 U 28 21 2abc 3a G Pᵒ

13:10 M III Y N 3 3 60 5 0 U 34 13* 2a Iᵒ

13:10 F V Y N 4 3 50 3 0 B 31 28 1a 2abc 3a Pᵈ

14:0 M V Y Y 5 6 10 6 5 U 52 0 2ac 3a Pᵈ

14:0 F V Y N 4 6 20 9 9 B 75 73 1abc 2abc 3ab Pᵈ Iᵒ

14:0 F V Y N 3 6 30 0 0 U 12 0* 2bc B G

14:2 F IV N N 3 2 70 1 1 B 25 24 1a 2abc 3a Pᵈ

14:4 F V Y N 3 2 70 0 0 B 26 21 1a 2abc No

14:6 F V Y N 4 6 20 0 1 U 18 17* 1a 2abc 3a Pᵈ

14:11 M V N Y 3 6 30 5 0 B 15 0 1a 2abc Pᵈ Iᵈ

15:1 F IV Y N 4 6 20 10 0 U 75 3 1ac 2abc B Pᵒ Iᵒ

15:2 M V Y Y 4 6 20 3 1 B 15 10 1ac 2abc 3a Pᵈ

15:2 M V Y N 4 4 40 4 2 B 38 0 1abc 2abc 3a B Pᵈ

15:2 M V N Y 4 3 50 4 2 B 92 74 2bc Pᵈ Iᵈ

15:5 M V N N 4 4 40 0 0 B 26 22 2abc B G

16:1 M IV Y N 3 3 60 0 0 B 31 26 2c No

16:6 M V Y Y 6 3 30 8 7 U 70 2 2abc 3a Pᵈ

16:7 M V Y N 4 3 50 2 2 U 33 21* 2bc 3a Pᵈ Iᵈ

Abbreviations: Age, y:mo, age in years and months. Gender: F, Female; M, Male. GMFCS, gross motor function classification system. Surgery, hip surgery: Y, Yes; N No. ITB, intrathecal baclofen therapy; Y, Yes; N, No. Characteristics of hip pain: raw score for intensity and frequency (0-6, 0-no pain). CHQ, Child Health Questionnaire hip pain score, scale 0 -100. Interference of hip pain on DA, daily activities, and sleep, numeric rating scale 0-10, 0-no interference. Side of hip pain: B, bilateral; U, unilateral. MP, migration percentage: MP max, the highest MP; MP min, the lowest MP; *, unilateral hip pain in hip with the lowest MP. Circumstances, circumstances of hip pain: provoked pain (1) a) hip mobilization/streching, b) palpation, c) weight bearing; pain linked to position (2) a) long time in same position b) at change of position c) during personal care; spontaneous pain (3) a) at night b) at cold weather. Medication last four weeks: B, Baclofen daily, G, gabapentin daily; pain medication: P, paracetamol, I, ibuprofen, N, naproxen; ᵈ, daily; ͦ, occasionally; No, no medication for spasticity or pain.
